# Supplementary material for: AMHconverter: an online tool for converting results between the different anti-Müllerian hormone assays of Roche Elecsys®, Beckman Access, and Kangrun
Source: PeerJ. 2023 Jun 5;11:e15301. doi: 10.7717/peerj.15301 (PMC10249628; doi:10.7717/peerj.15301)
Supplement: Supplemental Information 3 [file peerj-11-15301-s003.docx]

# The following are the results of fresh (day 0) and freeze-thaw samples tested on Kangrun platform:

| sample ID | Day-0 | Day-1 | Day-2 | Day-3 | Day-4 | Day-5 | Day-6 | Day-7 |
| --- | --- | --- | --- | --- | --- | --- | --- | --- |
| 1 | 12.89 | 13.19 | 12.82 | 13.52 | 13.27 | 13 | 12.58 | 12.98 |
| 2 | 20.36 | 21.15 | 19.71 | 19.61 | 20.58 | 21.22 | 20.14 | 19.9 |
| 3 | 5.86 | 5.68 | 5.8 | 5.66 | 5.76 | 5.61 | 5.69 | 6.14 |
| 4 | 10.18 | 10.26 | 9.67 | 10.39 | 10.43 | 9.76 | 10.09 | 10.52 |
| 5 | 2.25 | 2.22 | 2.24 | 2.32 | 2.18 | 2.25 | 2.21 | 2.19 |
| 6 | 1.24 | 1.28 | 1.3 | 1.28 | 1.28 | 1.24 | 1.2 | 1.22 |
| 7 | 0.62 | 0.63 | 0.59 | 0.61 | 0.64 | 0.59 | 0.6 | 0.62 |
| 8 | 4.69 | 4.65 | 4.72 | 4.78 | 4.62 | 4.8 | 4.61 | 4.91 |

The repeated measures ANOVA was used in our analysis. Eight time points were deemed as dummy variables and day-0 was used as reference, and the results of the analysis were as follows:


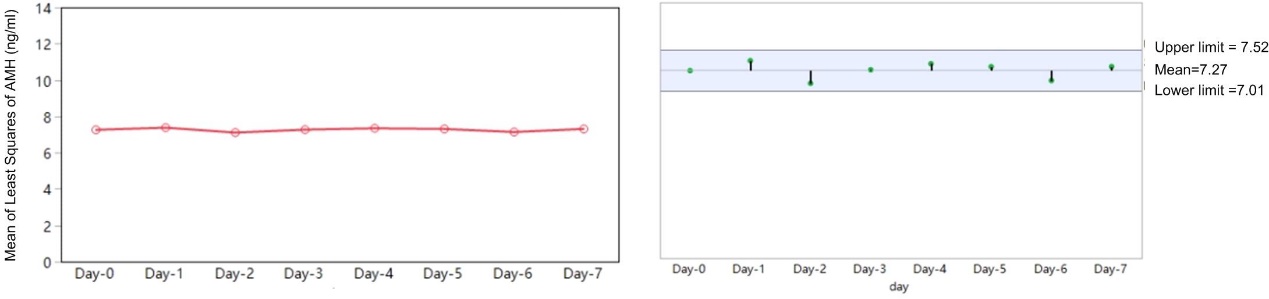


The result in the following table indicate that there is no statistical difference at each time point compared with day-0 (fresh samples).

|  | **Estimate** | **Standard error** | **denominator degrees of freedom** | **t ratio** | ***P*-value** | **95% lower limit of estimate** | **95% upper limit of estimate** |
| --- | --- | --- | --- | --- | --- | --- | --- |
| Intercept | 7.26125 | 2.417515 | 7.02 | 3.00 | 0.0198* | 1.5479996 | 12.9745 |
| Day-7 | 0.04875 | 0.137238 | 49.00 | 0.36 | 0.7239 | -0.227039 | 0.3245395 |
| Day-6 | -0.12125 | 0.137238 | 49.00 | -0.88 | 0.3813 | -0.397039 | 0.1545395 |
| Day-5 | 0.0475 | 0.137238 | 49.00 | 0.35 | 0.7307 | -0.228289 | 0.3232895 |
| Day-4 | 0.08375 | 0.137238 | 49.00 | 0.61 | 0.5445 | -0.192039 | 0.3595395 |
| Day-3 | 0.01 | 0.137238 | 49.00 | 0.07 | 0.9422 | -0.265789 | 0.2857895 |
| Day-2 | -0.155 | 0.137238 | 49.00 | -1.13 | 0.2642 | -0.430789 | 0.1207895 |
| Day-1 | 0.12125 | 0.137238 | 49.00 | 0.88 | 0.3813 | -0.154539 | 0.3970395 |

The results in the following table indicate that there is no statistical difference at each time point compared with the mean.

| **day** | **-day** | **difference** | **Standard error** | **95% Lower limit of difference** | **95% upper limit of difference** | **t ratio** |
| --- | --- | --- | --- | --- | --- | --- |
| Day-0 | mean | -0.004375 | 0.0907742 | -0.257018 | 0.2482681 | -0.05 |
| Day-1 | mean | 0.116875 | 0.0907742 | -0.135768 | 0.3695181 | 1.29 |
| Day-2 | mean | -0.159375 | 0.0907742 | -0.412018 | 0.0932681 | -1.76 |
| Day-3 | mean | 0.005625 | 0.0907742 | -0.247018 | 0.2582681 | 0.06 |
| Day-4 | mean | 0.079375 | 0.0907742 | -0.173268 | 0.3320181 | 0.87 |
| Day-5 | mean | 0.043125 | 0.0907742 | -0.209518 | 0.2957681 | 0.48 |
| Day-6 | mean | -0.125625 | 0.0907742 | -0.378268 | 0.1270181 | -1.38 |
| Day-7 | mean | 0.044375 | 0.0907742 | -0.208268 | 0.2970181 | 0.49 |

# Fresh and freeze-thaw sample data in Roche platform are as follows:

| ID | Day-0 | 6month |
| --- | --- | --- |
| 1 | 2.15 | 2.17 |
| 1 | 2.14 | 2.17 |
| 1 | 2.15 | 2.14 |
| 2 | 1.35 | 1.35 |
| 2 | 1.35 | 1.34 |
| 2 | 1.35 | 1.35 |
| 3 | 3.85 | 3.82 |
| 3 | 3.85 | 3.74 |
| 3 | 3.82 | 3.85 |
| 4 | 0.49 | 0.50 |
| 4 | 0.48 | 0.49 |
| 4 | 0.49 | 0.50 |
| 5 | 18.60 | 18.80 |
| 5 | 18.60 | 19.20 |
| 5 | 18.50 | 18.80 |
| 6 | 6.90 | 6.84 |
| 6 | 6.84 | 6.93 |
| 6 | 6.93 | 6.80 |
| 7 | 0.09 | 0.09 |
| 7 | 0.09 | 0.09 |
| 7 | 0.09 | 0.09 |

Using the same statistical method of repeated-measures ANOVA, results showed no statistically difference in AMH concentrations at -20℃ storage for 6 months compared to day-0 (fresh) samples. The statistical results are as follows:

|  | **Estimate** | **Standard error** | **Denominator degrees of freedom** | **t ratio** | ***P*-value** | **95% lower limit of estimate** | **95% upper limit of estimate** |
| --- | --- | --- | --- | --- | --- | --- | --- |
| intercept | 4.7667143 | 2.486053 | 6.00 | 1.92 | 0.1036 | -1.316088 | 10.849517 |
| 6 month | 0.0452381 | 0.054085 | 6.00 | 0.84 | 0.4350 | -0.087103 | 0.1775789 |

|  | **Estimate** | **Standard error** | **Degrees of freedom** | **95% upper limit of difference** | **t ratio** |
| --- | --- | --- | --- | --- | --- |
| 6 month | 4.8119524 | 2.4860526 | 6.0014 | -1.270850 | 10.894755 |
| Day-0 | 4.7667143 | 2.4860526 | 6.0014 | -1.316088 | 10.849517 |
